# Analysis of the potential of cancer cell lines to release tissue factor-containing microvesicles: correlation with tissue factor and PAR2 expression

**DOI:** 10.1186/s12959-016-0075-3

**Published:** 2016-01-19

**Authors:** Camille Ettelaie, Mary EW Collier, Sophie Featherby, Naima E. Benelhaj, John Greenman, Anthony Maraveyas

**Affiliations:** Biomedical Section, Department of Biological Sciences, University of Hull, Cottingham Road, Hull, HU6 7RX UK; Department of Cardiovascular Sciences, University of Leicester, Clinical Sciences Wing, Glenfield General Hospital, Leicester, LE3 9QP UK; Division of Cancer-Hull York Medical School, University of Hull, Cottingham Road, Hull, HU6 7RX UK

**Keywords:** Tissue factor, Microvesicles, PAR 2, Blood coagulation, Cell line

## Abstract

**Background:**

Despite the association of cancer-derived circulating tissue factor (TF)-containing microvesicles and hypercoagulable state, correlations with the incidence of thrombosis remain unclear.

**Methods:**

In this study the upregulation of TF release upon activation of various cancer cell lines, and the correlation with TF and PAR2 expression and/or activity was examined. Microvesicle release was induced by PAR2 activation in seventeen cell lines and released microvesicle density, microvesicle-associated TF activity, and phoshpatidylserine-mediated activity were measured. The time-course for TF release was monitored over 90 min in each cell line. In addition, TF mRNA expression, cellular TF protein and cell-surface TF activities were quantified. Moreover, the relative expression of PAR2 mRNA and cellular protein were analysed. Any correlations between the above parameters were examined by determining the Pearson’s correlation coefficients.

**Results:**

TF release as microvesicles peaked between 30–60 min post-activation in the majority of cell lines tested. The magnitude of the maximal TF release positively correlated with TF mRNA (c = 0.717; *p* < 0.001) and PAR2 mRNA (c = 0.770; *p* < 0.001) expressions while the percentage increase correlated with PAR2 mRNA (c = 0.601; *p* = 0.011) and protein (c = 0.714; *p* < 0.001). There was only a weak correlation between resting TF release, and microvesicle release. However, TF release in resting cells did not significantly correlate with any of the parameters examined. Furthermore, TF mRNA expression correlated with PAR2 mRNA expression (c = 0.745; *p* < 0.001).

**Discussion and Conclusions:**

In conclusion, our data suggest that TF and PAR2 mRNA, and PAR2 protein are better indicators of the ability of cancer cells to release TF and may constitute more accurate predictors of risk of thrombosis.

## Background

Cancer-related venous thromboembolism (VTE) is the second most frequent cause of cancer-related mortality and morbidity associated with many types of cancers. The hypercoagulable state is detectable in up to 70 % of cancer patients with a 2–8 fold higher risk of thromboembolism in cancer patients than normal individuals. VTE itself may also be an indicator of malignancy. One main cause of the increased procoagulant activity during malignancy is the increased expression of tissue factor (TF) by tumour cells [1, 2]. In the past, analyses of TF in cancers have mainly involved the measurement of TF antigen or activity on the surface, or within the cancer cells [3–7]. A more recent factor associated with the risk of thrombosis in cancer patients is the release of procoagulant tumour-derived microvesicles into the blood circulation. These microvesicles can harbour the protein TF acting as a major inducer of coagulation as well as containing phosphatidylserine, the presence of which is essential for the coagulation [8–10]. However, while elevated levels of these microvesicles are often associated with the hypercoagulable state [8–18], there is no clear association between the concentration of circulating microvesicles and the incidence of thromboembolism [19–21].

It is known that cells may release microvesicles upon activation and depending on the stimuli, these microvesicles may harbour TF [12, 22–28]. One such factor capable of inducing the release of TF as microvesicles is the activation of protease activated receptor 2 (PAR2) on the cell surface [24, 25] which itself, may participate in cancer progression [29, 30]. The activation of PAR2 may occur through the proteolytic activity of coagulation factor Xa and TF-factor VIIa complex [31]. The exposure of cancer cells to these proteases, for example as a consequence of coming into contact with blood, may therefore prompt dormant TF-bearing tumour cells to release large quantities of TF-bearing microvesicles. We hypothesise that the potential of tumour cells to release TF upon activation, may be a critical criterion in the promotion of hypercoagulable state and precipitation of thrombosis. In this in vitro study, we have examined this attribute which we have termed “TF-release potential”, in seventeen different cells lines, and by correlating this potential to relevant properties including TF and PAR2 expression, attempted to identify possible marker which may prove to be indicative of the risk of thrombosis during cancer in vivo.

## Methods

### Cell lines

Cells lines (ATCC, Teddington, UK) MDA-MB-231, MIA-Paca-2 and A375 were cultured in DMEM; BxPC-3, ASPC-1, T-47D, ZR-75-1, WM-266-4 and CorL23 lines were cultured in RPMI-1640; MCF-7, LS147T, CaCo-2 and SK-MEL-1 cells were cultured in EMEM; HT-29 and SKOV-3 cells were cultured in McCoy’s 5a medium; NCI-H209 cells were culture in Iscove medium and LoVo cells were cultured in Ham’s F-12 K medium. All media were obtained with Lonza (Cambridge, UK) contained foetal calf serum 10 % (v/v; Source Bioscience plc, Nottingham, UK) and MIA-PaCa-2 cells were also supplemented with 1 % (v/v) horse serum (Sigma Chemical Company Ltd., Poole, UK).

### Microvesicle isolation, analysis and quantification

Cells (2 × 10^5^) were seeded out in 6-well plates and permitted to adhere. All cells lines were washed and pre-adapted to respective serum-free medium prior to activation and harvesting of conditioned media. To induce microvesicle release, the cells were stimulated with PAR2-activating peptide (PAR2-AP); SLIGRL; (20 μM) (Sigma). The released cell-derived microvesicles were then isolated from conditioned media and resuspended in PBS according to published procedures [24, 25, 32–34]. The microvesicles were quantified using the Zymuphen MP assay kit (Hyphen BioMed/Quadratech Ltd, Epsom, UK) since this attribute was relevant to the functionality of the microvesicles and consequently, to this study.

### Quantification of TF and PAR2 mRNA expression

Total RNA was isolated using the TRI-reagent system (Sigma) from 5 × 10^5^ cells. To quantify the amount of TF mRNA, real-time RT-PCR was carried out in triplicates using primer sets designed to detect TF and β-actin and the absolute amounts of TF mRNA quantified as previously described [35]. Single-step RT-PCR was carried out in triplicates using 100 ng of total RNA from each sample tested. A set of previously prepared standard TF mRNA ranging 0.05-10 ng was included [35]. To assess the expression of PAR2, real-time RT-PCR was carried out in triplicates using 100 ng of total RNA from each sample using primer sets designed to PAR2 and β-actin mRNA. After the amplification, the ratios of PAR2 mRNA were determined with respect to that present in MIA-PaCa-2 cells which express low levels of PAR2, using the 2^-ΔΔCT^ method [36]. The reaction was carried out at an annealing temperature of 60 °C using the GoTaq® 1-Step RT-qPCR System (Promega Corporation Ltd, Southampton, UK) on an iCycler thermal cycler (Bio-Rad, Hemel Hempstead, UK) and the data analysed. The primers used were:

- TF-forward: 5'-TACAGACAGCCCGGTAGAGTG-3',
- TF-reverse: 5'-GAGTTCTCCTTCCAGCTCTGC-3',
- PAR2-forward: 5'-GAGCCATGTCTATGCCCTGT-3'
- PAR2-reverse: 5'-GACACTTCGGCAAAGGAGAG-3'
- β-actin-forward: 5'-TGATGGTGGGCATGGGTCAGA-3',
- β-actin-reverse: 5'-GTCGTCCCAGTTGGTGACGAT-3'

### Analysis of TF and PAR2 antigen and TF activity

To measure the TF content of the cells, non-activated cells (5 × 10^5^) were lysed in a non-denaturing lysis buffer (125 μl) containing protease inhibitors (Promega). The amount of TF protein was then quantified with a TF-antigen EIA kit (Enzyme Research Laboratories Ltd, Swansea, UK) using a recombinant full-length TF (Sekisui Diagnostics/Invitech Ltd, Molesworth, UK) as previously described [24, 32, 35]. The relative amounts of cellular PAR2 were assessed by lysing non-activated cells (5 × 10^5^) in Laemmeli’s buffer (150 μl). The samples were then separated by 12 % (w/v) SDS-PAGE and transferred to nitrocellulose membranes at 100 V for 1 h. The membranes were then blocked with TBST (10 mM Tris–HCl pH 7.4, 150 mM NaCl, 0.05 % Tween-20) and incubated overnight with a mouse monoclonal antibody against human PAR2 (SAM11; Santa Cruz Technology, Heidelberg, Germany) diluted 1:1000 (v/v) in TBST at 4 °C. The membranes were then washed with TBST and probed with a goat anti-mouse horse-radish peroxidase (HRP)-conjugated antibody (Santa Cruz) diluted 1:2000 (v/v) for 90 min. The PAR2 bands were then visualised using the TMB substrate for HRP (Promega) and recorded. The specificity of the anti-PAR2 antibody was confirmed by using human dermal endothelial cells as control cells; These cells exhibit a single band of around 50 kDa when probed the SAM11 antibody in western blot [37]. As loading controls, the level of GAPDH was measured using a goat anti-human GAPDH antibody (V-18; Santa Cruz) followed by a donkey anti-goat alkaline phosphatase-conjugated antibody (Santa Cruz) diluted 1:1000 (v/v) and incubated for 90 min. The bands were then visualised using the Western Blue stabilised alkaline phosphatase-substrate (Promega) and recorded.

Microvesicle-associated and cell surface (5 × 10^4^) TF activities were measured using a modified chromogenic thrombin-generation assay by inclusion of Tris-buffered saline (10 mM Tris–HCl pH 7.0, 154 mM NaCl) as described previously [33]. In addition, the TF-mediated factor Xa generation and factor VIIa activity were also measure using the Actichrome TF activity assay and SPECTROZYME^®^ FVIIa (Sekisui Diagnostics, Maidstone, UK). To demonstrate that the thrombin generation was TF-dependent, samples were pre-incubated with an inhibitory TF monoclonal antibody 4509 (Axis-Shield, Dundee, UK) (10 μg/ml) for 1 h prior to the thrombin-generation assay as before [24, 25, 32]. In addition, to ensure that the thrombin activation was factor VIIa-dependent, selected reactions were carried out in the presence of factor VIIa-deficient plasma as previously described [34].

### Statistical analysis

All data represent the calculated mean values from the number of experiments stated in each figure legend ± the calculated standard error of the mean. Statistical analysis was carried out using the Statistical Package for the Social Sciences (SPSS Inc. Chicago, USA). Statistically significant differences between groups were assessed by paired t-test. Correlations between the measured cellular attributes were examined by performing multiple Pearson’s correlation analysis together with the significance of the values.

## Results

The procedure for isolation of the microvesicles was validated previously and the lack of significant amounts of exosomes shown by measuring the presence of CD9 within the isolated microvesicle samples [33, 34].

### Comparison of the expression of TF mRNA and protein in cell lines

**Fig. 1:**
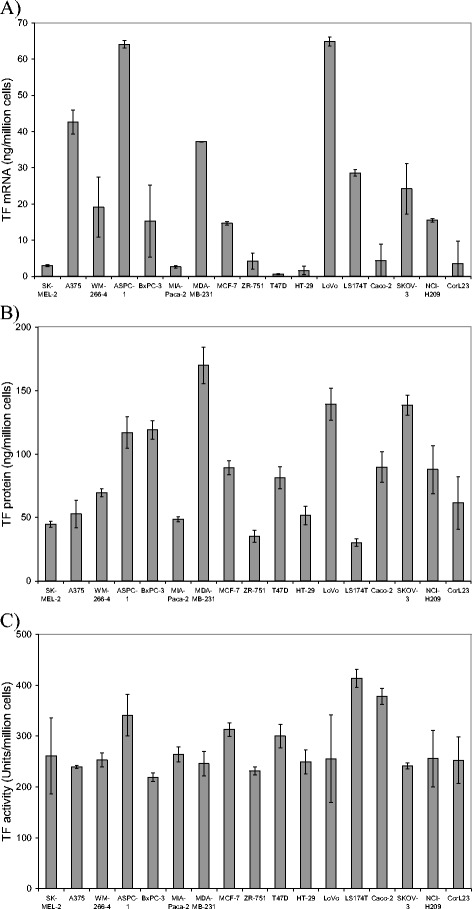
Quantitative analysis of TF mRNA, protein and surface activity of cell lines. **a** Total RNA was isolated from each cell lines (5 × 10^5^ cells) and 100 ng used to determine the absolute amount of TF mRNA in each sample using a quantitative real-time RT-PCR. (*n* = 3) **b** Cells (5 × 10^5^) were also lysed and TF protein quantified using a TF-antigen EIA kit. (*n* = 3) C) Cell surface (5 × 10^4^) TF activities were measured for each cell lines using a modified chromogenic thrombin-generation assay (*n* = 3)

The amount of TF mRNA from cell lines was quantified using a real-time RT-PCR procedure and the amplification of the standard TF mRNA was shown to be linear over the range of 0.05-10 ng (not shown) [35]. The basal expression of TF mRNA was highest in LoVo, ASPC-1, A375 and MDA-MB-231 cell lines, with moderate amounts being expressed in LS174T, SKOV-3, WM-266-4, NCI-H209, MCF-7 and BxPC-3 cells and low levels present in the remainder of the cell lines examined (Fig. 1a). Total cellular TF protein content moderately correlated (Table 1) with the level of mRNA expression (Pearson correlation coefficient c = 0.532; *p* = 0.028) and in general, cells expressing high levels of TF mRNA including LoVo, ASPC-1 and MDA-MB-231 cells also contained high TF antigen levels (Fig. 1b). Exceptions to this were cell lines such as T47D and CaCo-2 which produced moderate amounts of TF protein despite low level mRNA expression, and also cell lines such as A375 which contained moderate levels of the protein despite high level expression of TF mRNA. The observation for the lack correlation between TF mRNA and TF protein in some of the cell lines tested may arise from the differing rates of TF mRNA stability in these cells and may also be significant in the malignant properties of these cells. However, another mechanism that alters the correlation between these two measurements is the rate of release of TF from the cells, under resting conditions since this appears to differ in the cell lines tested. Furthermore, surface TF activity in resting cells varied between the cells with LS174T possessing almost twice as much activity as BxPc-3 cells (Fig. 1c), but did not correlate with TF mRNA or protein expression levels (Table 1). The lack of a strong correlation between TF activity and antigen is not surprising considering that TF activity is strongly altered by its status and the lipid components of the cell membrane domains with which TF associates [38–42]. Furthermore, the rate of TF transport and release as active microvesicles, is also likely to influence the perceived TF activity on the cell surface [35]. Attempts to measure TF activity using factor Xa generation assay and factor VIIa enzymatic activity did not produce sufficiently large differences and the data was abandoned in these cases.

**Table 1:** Correlations between cell line parameters

		TF mRNA	TF protein	Surface TF activity	MV-TF from resting cells	MV-TF from activated cells	Increase in MV-TF activity	PAR2 mRNA	PAR2 protein	Time-point of maximal TF release
TF mRNA	Pearson Correlation	-	0.532*	0.097	0.104	0.717^**^	0.341	0.745^**^	0.103	0.342
	Significance		0.028	0.710	0.691	0.001	0.181	0.001	0.695	0.179
TF protein	Pearson Correlation	0.532*	-	−0.175	0.094	0.507*	0.441	0.548*	0.341	0.501*
	Significance	0.028		0.503	0.720	0.038	0.077	0.023	0.180	0.041
Surface TF activity	Pearson Correlation	0.097	−0.175	-	0.209	0.160	−0.196	0.119	0.186	0.391
	Significance	0.710	0.503		0.420	0.539	0.451	0.650	0.475	0.121
MV-TF from resting cells	Pearson Correlation	0.104	0.094	0.209	-	0.483^*^	−0.453	0.033	−0.238	0.030
	Significance	0.691	0.720	0.420		0.050	0.053	0.899	0.358	0.908
MV-TF from activated cells	Pearson Correlation	0.717^**^	0.507*	0.160	0.483^*^	-	0.370	0.770^**^	0.242	0.484*
	Significance	0.001	0.038	0.539	0.050		0.144	<0.001	0.350	0.049
Increase in MV -TF Activity	Pearson Correlation	0.341	0.441	−0.196	−0.453	0.370	-	0.601^*^	0.714^**^	0.383
	Significance	0.181	0.077	0.451	0.053	0.144		0.011	0.001	0.129
PAR2 mRNA	Pearson Correlation	0.745^**^	0.548*	0.119	0.033	0.770^**^	0.601^*^	-	0.309	0.618*
	Significance	0.001	0.023	0.650	0.899	<0.001	0.011		0.227	0.008
PAR2 protein	Pearson Correlation	0.103	0.341	0.186	−0.238	0.242	0.714^**^	0.309	-	0.430
	Significance	0.695	0.180	0.475	0.358	0.350	0.001	0.227		0.085
Time-point of maximal TF release	Pearson Correlation	0.342	0.501*	0.391	0.030	0.484*	0.383	0.618*	0.430	-
	Significance	0.179	0.041	0.121	0.908	0.049	0.129	0.008	0.085	

**Fig. 2:**
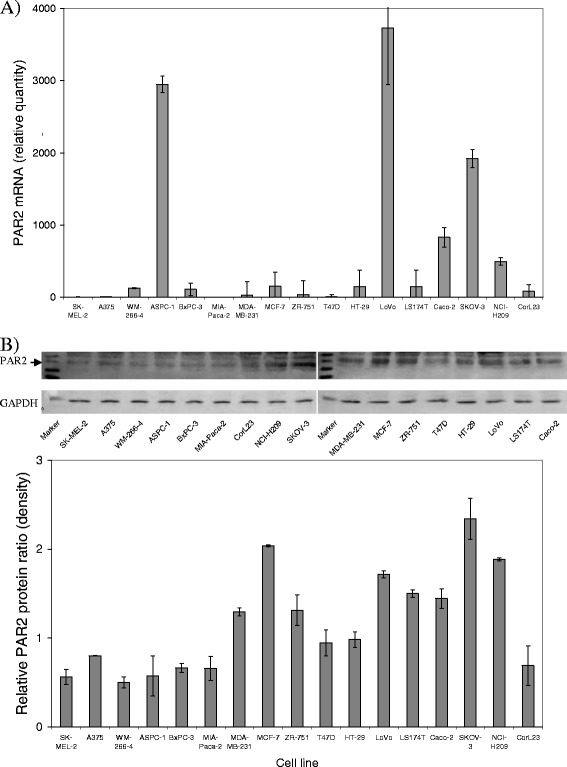
Semi-quantitative analysis of PAR2 mRNA and protein in cell lines. **a** Total RNA was isolated from each cell lines (5 × 10^5^ cells) and 100 ng used to determine the relative amount of PAR2 mRNA by real-time RT-PCR using β-actin mRNA as reference. The ratios of PAR2 mRNA were determined with respect to that expressed by MIA-PaCa-2 cells. (*n* = 3) **b** The relative amount of cellular PAR2 was assessed in lysed non-activated cells (5 × 10^5^) analysed by western blot and probed with an antibody against human PAR2 (SAM11) diluted 1:1000 (v/v) in TBST, developed with a goat anti-mouse HRP-conjugated antibody and visualised using the TMB substrate. The level of GAPDH was measured using an anti-human GAPDH antibody (V-18) followed by a donkey anti-goat alkaline phosphatase-conjugated antibody and visualised as above. (*n* = 3)

**Fig. 3:**
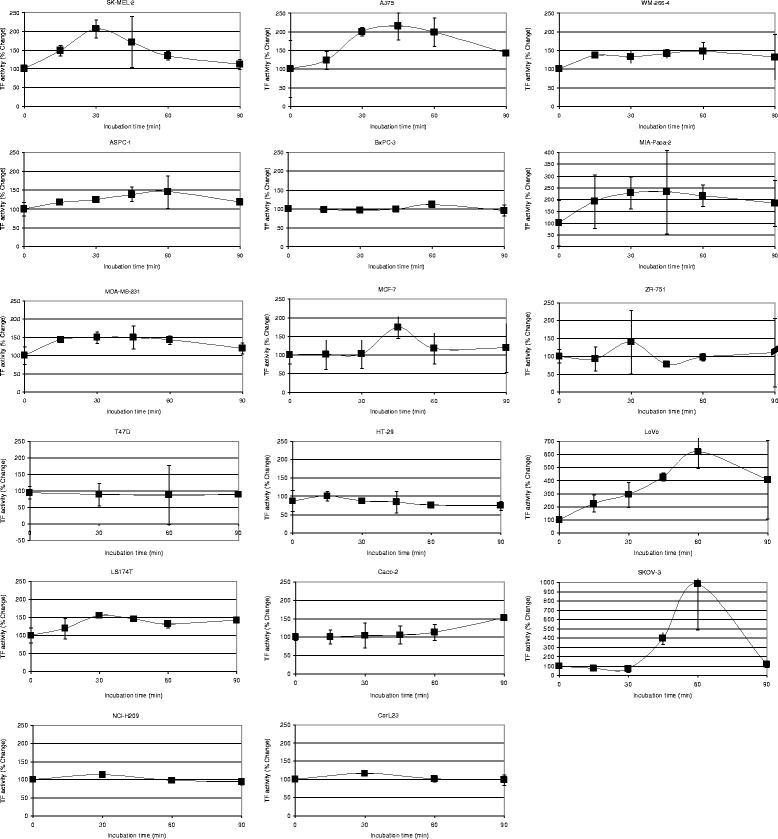
Time course of the microvesicle-associated TF activity released into the media by the cell lines. Cells (2 × 10^5^) were seeded out in 6-well plates and pre-adapted to respective serum-free medium. Microvesicle release was induced by incubation with PAR2-AP; SLIGRL; (20 μM). The released cell-derived microvesicles were isolated by ultracentrifugation at intervals up to 90 min and resuspended in Tris-saline. Microvesicle-associated TF activity was measured using the thrombin-generation assay, for each sample (*n* = 3)

**Fig. 4:**
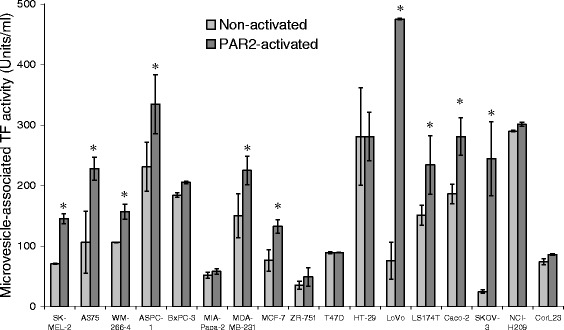
Analysis of minimal and maximal levels of released microvesicle-associated TF activity by the cell lines. Cells (2 × 10^5^) were seeded out in 6-well plates and pre-adapted to respective serum-free medium. Microvesicle release was induced by incubation with PAR2-AP; SLIGRL; (20 μM). The released cell-derived microvesicles were then isolated by ultracentrifugation prior to activation (resting), and at the interval with the maximum level of microvesicle-associated TF activity (see Fig. 3). The microvesicles were resuspended in Tris-saline and TF activity in each sample was measured using the thrombin-generation assay, in each sample. (*n* = 3; * = *p* < 0.05 vs. the respective non-activated sample) (*N/T* = Not tested)

Parameters associated with seventeen cell lines (shown on the table) were determined as shown in Figs. 1–4. Correlations between the measured cellular attributes were examined by performing multiple Pearson’s correlation analysis (*n* = 3; * = *p* < 0.05; ** = *p* < 0.01)

### Comparison of the level of PAR2 mRNA and antigen in cell lines

Due to the lack of a reliable source of in vitro-transcribed synthetic PAR2 mRNA, the levels of PAR2 expression were only measured semi-quantitatively and compared to that observed in MIA-PaCa-2 cell line which exhibited very little PAR2 expression. Analysis of PAR2 protein by western blot indicated the presence of up to 3 bands which is in line with those indicated on the manufacturer's site (Santa Cruz Biotechnology) and are thought to arise from the differential glycosylation of PAR2 protein by different cells [43]. The levels of PAR2 mRNA were highest in LoVo, ASPC-1 and SKOV-3 cell lines (Fig. 2) while the levels of PAR2 proteins were elevated in LoVo, SKOV-3, NCI-H209 and MCF-7 cells lines. The levels of PAR2 mRNA and protein correlated weakly with each other (c = 0.309; *p* = 0.227). Relative PAR2 mRNA levels correlated with TF mRNA expression (c = 0.745; *p* < 0.001) and protein levels (c = 0.548; *p* = 0.023). Neither PAR2 mRNA, nor protein levels correlated with the cell surface TF activity.

### Time course of TF release by the cell lines from PAR2-activated cells

This study investigated the possibility that the activation of PAR2 may be a main influence in the ability of cancer cells to upregulate the procoagulant potential within the circulation. Moreover, the measurement of TF antigen associated with microvesicles does not necessarily correlated with the procoagulant potential of the microvesicles [44]. Consequently, we measured the microvesicle-associated TF activity rather than TF antigen levels. In order to assess the time-course of TF release from cells following the activation, equal numbers of cells (2 × 10^5^) were adapted to serum-free media and then activated by incubation with PAR2-AP (20 μM). Conditioned media was collected at intervals up to 90 min, microvesicles isolated and TF activity examined as above. Time-course measurements were carried out to assess the velocity of cell response and determine the interval before maximal TF release. However, since the time-course measurements were carried out on separate occasions, no comparison of the magnitudes between the various cell lines was carried out at this stage. Therefore, the percentage ratio of TF activity with respect to time zero has been presented (Fig. 3). The duration of incubation before achieving maximal TF release was between 30–60 min in all cell lines except in HT-29 cells in which it was 15 min, and in CaCo-2 cells in which it was at least 90 min. This speed of response moderately correlated with the expression of TF protein (c = 0.501; *p* = 0.041), PAR2 mRNA (c = 0.618; *p* = 0.008) and to PAR2 protein (c = 0.430; *p* = 0.085).

### Comparison of TF release by the cell lines under resting condition and following PAR2-activation

Under resting conditions the level of TF release as microvesicles greatly varied between the cell lines examined with ASPC-1, HT-29 and NCI-H209 releasing high levels of TF while SKOV-3 cells being restrained (Fig. 4). The release of TF from the cells under resting conditions did not correlate with any of the parameters measured. Using the data generated from the time-course analysis (Fig. 3), microvesicles were isolated from the conditioned media of activated cell, at the time-points of maximal TF release for each cell line, and the TF activity measured. At the point of maximal release, microvesicle-associated TF activity was greatest in LoVo and ASPC-1 cells and lowest in MIA-PaCa-2 and ZR-751 cell lines (Fig. 4). This maximal TF-release potential strongly correlated with TF mRNA expression (c = 0.717; *p* < 0.001) and PAR2 mRNA expression (c = 0.770; *p* < 0.001), and to a lesser extent correlated with cellular TF protein levels (c = 0.507; *p* = 0.038) and the duration required for achieving maximal release (c = 0.484; *p* = 0.049). The maximum TF released also partially correlated with TF release from resting cells (c = 0.483; *p* = 0.050) but not the cell-surface TF activity (Table 1). Finally, the ability to up-regulate TF release (not shown), expressed as the increase in the TF release as a percentage of TF release in non-activated cells, correlated with both PAR2 mRNA expression (c = 0.601; *p* = 0.011) and PAR2 protein levels (c = 0.714; *p* < 0.001) and was inversely correlated to the level of TF release from non-activated cells (c = −0.453; *p* = 0.053).

### Comparison of microvesicle release by the cell lines under resting condition and following PAR2-activation

**Fig. 5:**
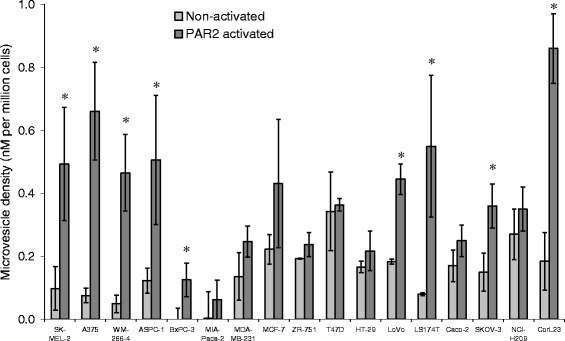
Analysis of minimal and maximal levels of released microvesicles by the cell lines. Cells (2 × 10^5^) were seeded out in 6-well plates and pre-adapted to respective serum-free medium. Microvesicle release was induced by incubation with PAR2-AP; SLIGRL; (20 μM) for the durations determined in Fig. 3. The released cell-derived microvesicles were then isolated by ultracentrifugation at the interval with the maximum level of microvesicle-associated TF activity (see Fig. 3). Samples of cells were also incubated for the same periods but without activation (resting) and microvesicles isolated. The microvesicles were resuspended in Tris-saline and the density in each sample determined using the Zymuphen MP assay kit. (*n* = 3; * = *p* < 0.05 vs. the respective non-activated sample)

Due to the nature of this investigation, the quantification of the microvesicles was carried out according to the functional properties of the microvesicles (i.e. phosphatidylserine content) rather that the physical quantities of the microvesicles (number and size distribution). The level of microvesicles release varied greatly between the cell lines (Fig. 5) and the greatest increase in microvesicle release was observed in CorL23, A375 and LS174T cell lines. Neither the density of microvesicle released after activation, nor the increase in the microvesicle density correlated with any of the other parameters. Furthermore, the release of microvesicles under resting conditions only correlated weakly with the amount of cellular PAR2 protein (c = 0.441; *p* = 0.100).

## Discussion

The association between cancer and thrombosis has long been established and many of the factors linking these conditions have been characterised [1, 16, 45]. However, parallel associations between procoagulant properties of cancer cells and the induction of blood coagulation do not appear to be sufficiently significant to permit the prognostic determination of the risk of thrombosis [4, 6]. The ability of cancer cells to express high levels of TF is well established [2] but the examination of the level of total and surface TF antigen, or cell-surface TF activity does not present a clear and definitive correlation with risk of clot formation [5, 6, 19–21]. In addition, while there has been a recent upsurge in studies examining the association of TF-bearing microvesicles and incidence of thromboembolism, correlations arising from these data have been reported to be heterogeneous [14, 45]. It has been demonstrated that released microvesicles are rapidly cleared by cellular uptake in vitro and from the bloodstream in vivo, with estimates ranging from minutes to a few hours [33, 46–51]. Consequently, any TF released as tumour-derived microvesicles may not remain within the circulation for long enough to accumulate to levels capable of promoting clot formation while concurrently, TF-bearing microvesicles may be present at measurable levels at the time of sampling. One feature of many cells is the ability to release TF as microvesicles in bursts upon activation of the cell. Such bursts in TF release into the bloodstream may fluctuate in duration and magnitude and may also vary in the length of time before the onset of the release of TF-bearing microvesicle [16, 52, 53]. Therefore, in this study we examined the ability of seventeen cancer cell lines spanning various tissues to release TF in response to PAR2 activation. We used PAR2 activation as a stimulus for the study since 1) the release of TF-bearing microvesicles occurs in a much shorter time than for example stimulation with TNFα [54] and also, remained unaltered in the untreated control cells. 2) PAR2 activation represents a more controlled activation of cells without influencing other pro-inflammatory mechanisms within the cell as observed with LPS treatment. Together, these advantages permit the analysis of TF release without the influences arising from de novo expression of various genes which may complicate the analysis. Finally, 3) we hypothesise that the exposure of tumour cells to bloodstream may induce low level generation of factor Xa and TF-factor VIIa complex allowing for the activation of PAR2 on cancer cells, without substantial amounts of clot formation within the tumour cells’ immediate locality.

Activation of cells generally resulted in the upregulation of TF release within 30–60 min but varied hugely in magnitude in the cell lines tested. Interestingly, the change in the rate of TF release in activated cells was only moderately dependent on the rate of TF release while under resting conditions, or the amount of TF protein stored within the cells. Previously, we showed that the suppression of TF expression in five cell lines resulted in divergent rates of decline in the amount of cellular TF [35]. This was attributed to the background rate of TF release from these cells indicating that TF reserves were depleted at a faster rate when the amount of microvesicle-associated TF was maintained by the cells. In fact the percentage increase in the TF release following activation appeared to be inversely correlated to the ability of cells to release TF under resting conditions. Therefore, the level of cellular TF protein stored within the cells may be a function of the turnover of TF, determined by both the expression of TF mRNA and TF protein release from the cells and hence, only partly correlates with TF mRNA expression. This also explains the heterogeneous correlation between the level of circulating TF containing microvesicles and the incidence of thrombosis [14, 45] since this correlation may be positive or negative, depending on whether the tumour cells are activated at the time of sampling. In addition, a weak correlation between cell surface TF activity and microvesicle-associated TF activity was detected in resting cells. This is also in agreement with the notion that TF is transferred to the cell surface prior to release as microvesicle although, the control of TF activity at the cell surface may strongly be regulated by TF encryption [4]. In agreement with the above hypothesis, the level of microvesicle-associated TF during the short-term burst in TF release would be dependent on the ability of cell to replenish TF reserves through mRNA expression and therefore strongly correlated with the TF mRNA expression levels. Furthermore, the magnitude of TF release correlated strongly with the PAR2 mRNA expression in the cells. Interestingly, the level of TF mRNA expression also strongly correlated with the expression of PAR2 mRNA but not PAR2 protein. However, the turnover of PAR2 includes internalisation, recycling and degradation which may alter the perceived level of available PAR2 protein. The induction of TF expression following PAR2 activation has been shown previously [55]. Therefore, low level activation of PAR2 may also contribute to the ability of cells to replenish TF reserves and enhance the cellular “TF-release potential” through separate mechanisms involving the upregulation of TF gene expression. However, the change in TF release, as a percentage of the levels observed in resting cells appears to be related to PAR2 mRNA and PAR2 protein levels. Perhaps this is not surprising in our experiments since the cells were activated by incubation with PAR-activating peptide. However, it is surprising that 1) the strongest correlation was observed between TF release and PAR2 mRNA expression and 2) some cell lines such a ZR-75-1 did not respond proportionally to PAR2 activation. In contrast, the phosphorylation and the subsequent release of TF is not dependent of PAR1 activation [24, 56]. Although, the release of microvesicles themselves may occur upon activation of PAR1 and PAR2 by separate mechanisms [57]. In our present study, we did not observe a significant correlation between microvesicle release and TF release, although in general a higher TF release was often accompanied with moderate to high microvesicle release. Therefore, it is unlikely that PAR1 activation would have any direct influence on TF release potential although indirect mechanisms have not been ruled out. Additionally, since glycosylation of PAR2 may alter its trafficking and function, the patterns observed in Fig. 2 may hold further clues to the "TF-release potential" property of the cell lines and need further investigation.

## Conclusion

Our cohort of cell lines used in this study was not sufficiently large to merit the inference of attributes to particular types of cancer and as such, we have avoided such speculation. However, more extensive studies of these parameter may provide characteristic trends in different cancer types. In conclusion, this study is consistent with previous reports and suggests that while PAR2 may maintain the level of TF expression [55] this does not constitute a superior biomarker than measuring TF itself. In contrast, the ability of cells to release TF as cell-derived microvesicles may be upregulated rapidly and significantly following the activation of PAR2. Therefore, quantification of the levels of TF and PAR2 mRNA and possibly PAR2 protein, may prove to be a means of determining the potential of cancer cells to release TF-containing microvesicles and constitute a more accurate predictor of risk of thrombosis in vivo.

### Consent for publication

Not applicable.
